# Genetic variability of immune checkpoints in asbestos-related diseases

**DOI:** 10.2478/raon-2026-0035

**Published:** 2026-06-26

**Authors:** Irma Zeljkovic, Katja Goricar, Tanja Blagus, Alenka Franko, Viljem Kovac, Vita Dolzan

**Affiliations:** 1 Faculty of Chemistry and Chemical Technology, University of Ljubljana, Ljubljana, Slovenia; 2 Biotechnical Faculty, University of Ljubljana, Ljubljana, Slovenia; 3 Pharmacogenetics Laboratory, Institute of Biochemistry and Molecular Genetics, Faculty of Medicine, University of Ljubljana, Ljubljana, Slovenia; 4 Clinical Institute of Occupational Medicine, University Medical Centre Ljubljana, Ljubljana, Slovenia; 5 Faculty of Medicine, University of Ljubljana, Ljubljana, Slovenia; 6 Institute of Oncology Ljubljana, Ljubljana, Slovenia

**Keywords:** asbestos, malignant mesothelioma, immune checkpoints, single nucleotide polymorphism

## Abstract

**Background:**

Asbestos exposure is associated with asbestos-related diseases such as pleural plaques, asbestosis, lung cancer, malignant mesothelioma (MM), as well as several other types of cancer. Although the exact mechanism underlying the development of asbestos-related diseases is not yet fully understood, the immune system may play an important role. Immune checkpoints such as programmed cell death receptor 1 (PD-1), programmed cell death ligand-1 (PD-L1), and cytotoxic T lymphocyte-associated protein 4 (CTLA-4) regulate immune responses and are crucial for maintaining immune tolerance, while defects in these mechanisms can lead to the development of cancer. The aim of present study was to investigate the association of polymorphisms in the immune checkpoint genes for PD-1 (*PDCD1*), PD-L1 (*CD274*), and CTLA-4 (*CTLA4*) with the risk of asbestos-related diseases.

**Subjects and methods:**

We conducted a retrospective case-control study. The cases included individuals with asbestosis or pleural plaques and MM patients. The controls were asbestos-exposed subjects who did not develop asbestos-related diseases. All subjects were genotyped for *PDCD1* (rs2227982, rs222798, rs10204525), *CD274* (rs2297136, rs4143815, rs4742098) and *CTLA4* (rs5742909, rs4553808, rs231775) polymorphisms using competitive allele-specific PCR. Statistical analysis was performed using logistic regression.

**Results:**

Our study showed that the *CTLA4* rs4553808 polymorphism was associated with a decreased risk of asbestosrelated diseases (OR = 0.34, 95% CI = 0.15-0.74, P = 0.007). Homozygous carriers of polymorphic rs4553808 G allele had a lower risk of developing pleural plaques as well as MM compared to the control group (OR = 0.28, 95% CI = 0.11-0.68, P = 0.01, and OR = 0.38, 95% CI = 0.16 -0.93, P = 0.03, respectively). However, carriers of polymorphic *CTLA4* rs231775 GG genotype had a higher risk of developing MM compared to the group of subjects with pleural plaques (OR = 1.79, 95% CI = 1.10-2.89, P = 0.02). The other investigated polymorphisms were not associated with any of the asbestos-related diseases.

**Conclusions:**

*CTLA4* polymorphisms may play a role in the susceptibility for asbestos-related diseases. Our results may contribute to a better understanding of the pathogenesis and progression of asbestos-related diseases.

## Introduction

Occupational or environmental asbestos exposure is the major risk factor for the development of many diseases, including pleural plaques, asbestosis, lung cancer, and malignant mesothelioma (MM), as well as several other types of cancer. Although asbestos has been banned in over 50 countries worldwide, the incidence of asbestos related diseases is still on the rise is some countries due to the long latency period between the exposure and disease occurrence.^1,2^

Pleural plaques are nonmalignant thickening on the pleura, often asymmetrical and bilateral and may develop up to 20 to 30 years after asbestos exposure in 50% of asbestos exposed workers. Pleural plaques are often asymptomatic and cause a slight lung function impairment when they grow. In the past, pleural plaques were referred to as a marker of asbestos exposure. However, in the recent years some studies have shown that they can indicate increased risk of asbestosis and MM.^2,3,4^

Asbestosis is one of the most common diseases related to asbestos exposure. It is an interstitial lung disease that progresses to diffuse pulmonary fibrosis after a long latency period. The disease is not reversable, and it continues to progress even after even after the end of asbestos exposure. The symptoms of the diseases are often non-specific like dyspnea, dry irritating cough and chest pain. The changes in pulmonary function are initially due to restrictive impairment and later due to obstructive air impairment. Asbestosis is also associated with a higher risk of lung cancer.^1,5^

MM is a rare but very aggressive cancer of the serous membranes and the most fatal asbestos-related disease.^6^ MM is found on the mesothelium, most commonly on pleura (65%), peritoneum (30%) or other serosal membranes (1%).^2^ The most common symptoms of pleural MM are dyspnea, chest pain, dry cough, and fatigue.^7^ Due to its non-specific symptoms, MM is often diagnosed in later stages, when the treatment is less effective. MM is treated by surgery in combination with chemotherapy and radiotherapy. In the latest years, the immunotherapy treatment of MM has also been extensively used.^8,9^

Studies have proposed that inflammation caused by asbestos is involved in pathogenesis of all asbestos-related diseases.^5,10^ After inhalation, asbestos fibers can accumulate in various tissues, including mesothelial cells in the pleural space, but also in other tissues such as the lung parenchyma, pericardium, and lymph nodes. This leads to local inflammatory response and increase in proliferation of mesothelial cells. Due to increase in metabolic activity, mesothelial cells orchestrate the production of local inflammatory mediators such as cytokines and growth factors. That leads to recruitment of leukocytes and enhancing inflam mation.^11^

The immune response is controlled by immune checkpoints inhibitors that enable effective immune response and immune tolerance. There are two types of immune checkpoints: stimulatory checkpoints that promote activation of naïve T cells and effector, memory and regulatory T cell response and inhibitory checkpoints that limit the threshold of T cell activation and duration of immune response.^12^

Programed death 1 (PD-1) receptor is an inhibitory immune checkpoint. PD-1 is constitutively expressed on T cells in thymus where it is required for their normal selection. PD1 is also found on activated T cells, B cells, monocytes, natural killer cells and dendritic cells. Program death ligand (PD-L1) expressed on cancer cells binds to PD-1 and activates pathways that suppress proliferation and survival of T cells.^13-15^

The cytotoxic T-lymphocyte-associated antigen 4 (CTLA-4) is also an inhibitory immune checkpoint. CTLA-4 is a CD28 homolog. The latter is a costimulatory receptor required for T cell activation. Both CTLA-4 and CD28 bind to the same ligands B7-1 and B7-2, however, CTLA-4 has a much higher binding affinity. As a result, CTLA-4 prevents T cell activation with competitive binding.^15,16^ Regulatory T cells constitutionally expressed on regulatory T cells (T_reg_) and it is crucial for their immunosuppressive role. T_reg_ cells are commonly infiltrated in tumor microenvironment and play immunosuppressive role with CTLA-4.^12^

Single nucleotide polymorphisms (SNPs) may have an influence on protein expression and function of proteins. Several studies have evaluated the impact of SNPs in genes coding for immune checkpoints PD-1 (*PDCD1*), PD-L1 (*CD274*), and CTLA-4 (*CTLA4*) and risk of developing different types of cancer.^17-19^ However, to the best of our knowledge the role of *PDCD1, CD274* and *CTLA4* genetic variability in the development of asbestos-related diseases has not been studied so far.

The aim of our study was to investigate the associations of *PDCD1, CD274* and *CTLA4* polymorphisms with the risk of developing asbestos-related diseases.

## Subjects and methods

### Study population

Our retrospective study included 902 subjects: 376 subjects with pleural plaques, 151 subjects with asbestosis, and 297 subjects with MM, as well as 78 subjects that were occupationally exposed to asbestos but did not develop any asbestos-related disease (controls)

Subject with pleural plaques, subject with asbestosis, and controls were selected from the cohort of workers with occupational exposure to asbestos. They were evaluated by the State Board for the Recognition of Occupational Asbestos Diseases at the Clinical Institute of Occupational, Traffic, and Sports Medicine in Ljubljana between 1.1.1999 and 31.12.2003. The diagnosis of all subjects was based on the Helsinki Criteria for Diagnosis^20^ and Attribution of Asbestos Diseases and the American Thoracic Society recommendations.^21^ In 2018, a follow-up was performed for all the subjects to confirm they did not develop any other asbestos-related disease. Patients with MM that were included in our study were treated at the Institute of Oncology Ljubljana between 1.11.2001 and 30.9.2018. The diagnosis of MM was performed by thoracoscopy in patients with pleural type of disease, or laparoscopy in patients with peritoneal type of disease and was histologically confirmed. For all subjects, demographic and clinical data were obtained from the medical records or during an interview.

The study was approved by the National Ethic Committee of Republic of Slovenia (31/07/04, 39/04/06 and 41/02/09) and was carried out according to the Declaration of Helsinki. All subjects provided written informed consent.

### Bioinformatic analysis

Bioinformatic analysis, in which we identified common immune check points SNPs, was carried out using Linkage Disequilibrium (LD) tag SNP Selection. We identified SNPs in the gene regions coding for immune checkpoints PD-1 (*PDCD1*), PD-L1 (*CD274*), and CTLA-4 (*CTLA4*) that have minor allele frequency (MAF) above 5% in European population. Function prediction was performed *in silico* using SNP Function Prediction Tool.^22^ Linkage disequilibrium (LD) between SNPs in one gene was carried out using LD link tool.^23^

### DNA extraction and genotyping

Genomic DNA was extracted from peripheral venous blood samples using MagMAX™ DNA Multi-Sample Kit (Applied Biosystems, Foster City, California, USA). The genotyping of the investigated *PDCD1, CD274* and *CTLA4* polymorphisms was performed by using a fluorescence-based competitive allele-specific polymerase chain reaction (KASP) assay, according to the manufacturer’s instructions (LGC Genomics, UK).

### Statistical analysis

Descriptive statistics were used to describe the study population. Continuous variables were presented as medians with 25%–75% range, while categorical variables were presented as frequencies. Differences between groups were assessed using Fisher’s exact test for categorical variables, including sex and smoking status, and the Kruskal-Wallis test for continuous variables, including age.

Deviation from Hardy-Weinberg equilibrium was evaluated for each investigated polymorphism using the chi-square test. Each polymorphism was analyzed under both additive and dominant genetic models. Univariate logistic regression analysis was used to compare genotype frequencies between groups and to estimate odds ratios (ORs) and 95% confidence intervals (CIs). The association of demographic parameters with asbestos-related diseases was also evaluated using univariate logistic regression. Parameters showing a significant association were subsequently included in multivariate logistic regression models.

The level of significance for all tests was set at 0.05 for all analyses. The statistical analyses were performed using IBM SPSS Statistics version 27.0 (IBM Corporation, Armonk, NY, USA). To assess the combined effect of all SNPs within the same gene, we reconstructed haplotypes using Thesias software.^24^

## Results

### Subjects’ characteristics

The characteristics of each subject group are presented in Table 1. Median age of patients with MM was 66 years and they were older than all the other groups (P < 0.001). There were no significant differences in sex (P = 0.214) and smoking (P = 0.325) between groups.

**TABLE 1.** Clinical characteristics of the subjects included in the study

Characteristic	Category/unit	No disease (N = 78)	Pleural plaques (N = 376)	Asbestosis (N = 151)	MM (N = 297)	P
Sex	Male N (%)	57 (73.1)	259 (68.9)	117 (77.5)	219 (73.7)	0.214^a^
	Female N (%)	21 (26.9)	117 (31.1)	34 (22.5)	78 (26.3)	
Age	Median (25%–75%)	53.2 (47.7–59.5)	54.9 (48.8–62.7)	59.1 (51.2–66.0)	66.0 (59.5–73.0)	<0.001^b^
Smoking	No N (%)	43 (55.1)	187 (49.7)	73 (48.3)	161 (55.7) [8]	0.325^a^
	Yes N (%)	35 (44.9)	189 (50.3)	78 (51.7)	128 (44.3)	

1 The number of missing data is presented in [].

a Fisher exact test;

b Kruskal Wallis test; MM - malignant mesothelioma

Among patients with MM, 263 (88.9%) patients had pleural MM, while 33 (11%) had peritoneal MM. Location of MM was not determined in 1 patient. The most common histological type of MM among subject was epithelioid type 223 (75.1%), however, 26 (8.8.%) of patients had biphasic MM and 21 (9.1%) patients had sarcomatoid MM. Regarding stage of cancer, 20 (7.6%) patients hade stage I, 63 (24.0%) had stage II, 87 (33.1%) had stage III and 93 (35.4%) had stage IV MM. According to Eastern Cooperative Oncology Group (ECOG) performance status, 18 (6.1%) patients had score 0, 150 (50.7 %) score 1, 110 (37.1%) score 2, 18 (6.1%) score 3. For 1 patient ECOG performance score could not be determined.

### Bioinformatic analyses

Using bioinformatic analysis, we identified nine polymorphisms that could have an impact on gene expression or protein levels or activity. Three were located in *PDCD1* (rs2227982, rs222798, rs10204525), three in *CD274* (rs2297136, rs4143815, s4742098), and three in *CTLA4* (rs5742909, rs4553808, rs231775). More detailed information on the results of bioinformatic analyses and the predicted functional effects of the selected SNPs is presented in Supplementary Figure 1 and Supplementary Table 1.

### Association of selected SNPs with susceptibility to asbestos-related diseases

Allele frequencies distributions in the investigated subject groups are presented in Table 2. In the whole study group, we evaluated if selected polymorphisms were associated with risk for any asbestos-related disease in comparison to subjects exposed to asbestos but did not develop any asbestos-related disease. The results are shown in Table 3. The carriers of two polymorphic *CTLA4* rs4553808 G alleles (GG genotype) were less susceptible to developing any asbestos related disease (OR = 0.34, 95% CI = 0.15-0.74, P = 0.007), the association was also significant after adjustment for age (OR = 0.32, 95% CI = 0.15-0.72, P = 0.006). None of the other investigated SNPs were significantly associated with susceptibility to any asbestos disease.

**TABLE 2.** Allele frequencies in the investigated subject groups

Protein (Gene)	SNP	Genotype	Controls	Pleural plaques	Asbestosis	MM
			N (%)	N (%)	N (%)	N (%)
PD-1 (*PDCD1*)	rs2227982	CC	74 (94.9)	356 (95.4)	144 (95.4)	287 (96.6)
		CT	4 (5.1)	17 (4.6)	7 (4.6)	9 (3.0)
		TT	0 (0.0)	0 (0.0)	0 (0.0)	1 (0.3)
		CT+TT	4 (5.1)	17 (4.6)	7 (4.6)	10 (3.4)
PD-1 (*PDCD1*)	rs2227981	CC	28 (36.4)	112 (30.1)	54 (35.8)	95 (32.0)
		CT	34 (44.2)	191 (51.3)	72 (47.7)	150 (50.5)
		TT	15 (19.5)	69 (18.5)	25 (16.6)	52 (17.5)
		CT+TT	49 (63.6)	260 (69.9)	97 (64.2)	202 (68.0)
PD-1 (*PDCD1*)	rs10204525	GG	59 (77.6)	295 (78.5)	120 (79.5)	248 (83.5)
		GA	15 (19.7)	75 (19.9)	30 (19.9)	48 (16.2)
		AA	2 (2.6)	6 (1.6)	1 (0.7)	1 (0.3)
		GA+AA	17 (22.4)	81 (21.5)	31 (20.5)	49 (16.5)
PD-L1 (*CD274*)	rs2297136	GG	15 (19.2)	77 (20.8)	41 (27.2)	54 (18.2)
		GA	43 (55.1)	180 (48.5)	72 (47.7)	169 (56.9)
		AA	20 (25.6)	114 (30.7)	38 (25.2)	74 (24.9)
		GA+AA	63 (80.8)	294 (79.2)	110 (72.8)	243 (81.8)
PD-L1 (*CD274*)	rs4143815	GG	39 (50.0)	159 (42.3)	65 (43.0)	134 (45.1)
		GC	32 (41.0)	162 (43.1)	66 (43.7)	135 (45.5)
		CC	7 (9.0)	55 (14.6)	20 (13.2)	28 (9.4)
		GC+CC	39 (50.0)	217 (57.7)	86 (57.0)	163 (54.9)
PD-L1 (*CD274*)	rs4742098	AA	43 (55.8)	189 (50.30)	76 (50.7)	160 (53.9)
		AG	27 (35.1)	147 (39.1)	64 (42.7)	117 (39.4)
		GG	7 (9.1)	40 (10.6)	10 (6.7)	20 (6.7)
		AG+GG	34 (44.2)	187 (49.7)	74 (49.3)	137 (46.1)
CTLA-4 *(CTLA4*)	rs4553808	AA	40 (51.9)	240 (63.8)	91 (60.3)	186 (62.8)
		AG	28 (36.4)	121 (32.2)	52 (34.4)	94 (31.8)
		GG	9 (11.7)	15 (4)	8 (5.3)	16 (5.4)
		AG+GG	37 (48.1)	136 (36.2)	60 (39.7)	110 (37.2)
CTLA-4 *(CTLA4*)	rs5742909	CC	54 (78.3)	302 (81.8)	117 (78.5)	237 (80.1)
		CT	13 (18.8)	63 (17.1)	28 (18.8)	54 (18.2)
		TT	2 (2.9)	4 (1.1)	4 (2.7)	5 (1.7)
		CT+TT	15 (21.7)	67 (18.2)	32 (21.5)	59 (19.9)
CTLA-4 *(CTLA4*)	rs231775	AA	39 (50.0)	181 (48.1)	71 (47.0)	128 (43.2)
		AG	28 (35.9)	157 (41.8)	65 (43.0)	120 (40.5)
		GG	11 (14.1)	38 (10.1)	15 (9.9)	48 (16.2)
		AG+GG	39 (50.0)	195 (51.4)	80 (53.0)	168 (56.8)

1 MM = malignant mesothelioma; SNP = single nucleotide polymorphism

**TABLE 3.** Comparison of genotype frequencies distributions in all patients and in patients with pleural plaques versus controls

Protein (Gene)	Genotype	All asbestos diseases	P	OR (95% CI)_adj_	P_adj_	Pleural plaques	P	OR (95% CI)_adj_	P_adj_
		OR (95% CI)				OR (95% CI)			
PD-1 (*PDCD1*)	CC	Reference		Reference		reference	reference	reference	reference
rs2227982	CT+TT	0.80 (0.276-2.31)	0.679	0.93 (0.32-2.72)	0.889	0.88 (0.29-2.70)	0.83	0.93 (0.30-2.86)	0.91
PD-1 (*PDCD1*)	CC	Reference		Reference		reference	reference	reference	reference
rs2227981	CT	1.30 (0.77-2.20)	0.322	1.27 (0.75-2.16)	0.371	1.40 (0.81-2.44)	0.23	1.41 (0.81-2.44)	0.23
	TT	1.04 (0.54-2.02)	0.898	1.01 (0.52-1.97)	0.971	1.15 (0.57-2.31)	0.69	1.14 (0.67-2.29)	0.71
	CT+TT	1.22 (0.75-1.99)	0.416	1.19 (0.73-1.95)	0.482	1.33 (0.79-2.22)	0.28	1.32 (0.79-2.22)	0.29
PD-1 (*PDCD1*)	GG	Reference		Reference		reference	reference	reference	reference
rs10204525	GA	0.91 (0.51-1.64)	0.749	0.91 (0.50-1.64)	0.737	1.00 (0.54-1.86)	1.00	0.99 (0.53-1.85)	0.98
	AA	0.36 (0.74-1.72)	0.198	0.44 (0.90-2.13)	0.304	0.60 (0.12-3.05)	0.54	0.62 (0.12-3.16)	0.57
	GA+AA	0.84 (0.48-1.49)	0.554	0.85 (0.48-1.50)	0.572	0.95 (0.53-1.72)	0.87	0.95 (0.53-1.72)	0.87
PD-L1 (*CD274*)	GG	Reference		Reference		reference	reference	reference	reference
rs2297136	GA	0.86 (0.46-1.58)	0.614	0.78 (0.42-1.46)	0.443	0.82 (0.43-1.56)	0.54	0.80 (0.42-1.53)	0.50
	AA	0.99 (0.49-1.98)	0.967	0.90 (0.44-1.83)	0.771	1.1 (0.54-2.30)	0.78	1.08 (0.52-2.24)	0.85
	GA+AA	0.90 (0.5-1.61)	0.713	0.82 (0.45-1.49)	0.516	0.91 (0.49-1.68)	0.76	0.89 (0.48-1.65)	0.70
PD-L1 (*CD274*)	GG	Reference		Reference		reference	reference	reference	reference
rs4143815	GC	1.24 (0.76-2.02)	0.397	1.20 (0.73-1.97)	0.468	1.24 (0.74-2.08)	0.41	1.23 (0.73-2.06)	0.43
	CC	1.60 (0.70-3.70)	0.267	1.67 (0.72-3.87)	0.232	1.93 (0.82-4.56)	0.14	1.92 (0.81-4.55)	0.14
	GC+CC	1.30 (0.82-2.07)	0.266	1.29 (0.80-2.06)	0.298	1.37 (0.84-2.23)	0.21	1.36 (0.83-2.21)	0.22
PD-L1 (*CD274*)	AA	Reference		Reference		reference	reference	reference	reference
rs4742098	AG	1.23 (0.74-2.03)	0.421	1.21 (0.73-2.01)	0.446	1.24 (0.73-2.10)	0.43	1.23 (0.73-2.09)	0.44
	GG	1.01 (0.44-2.34)	0.978	1.00 (0.43-2.34)	0.993	1.30 (0.55-3.10)	0.56	1.29 (0.54-3.07)	0.57
	AG+GG	1.18 (0.74-1.90)	0.481	1.17 (0.73-1.87)	0.527	1.25 (0.76-2.05)	0.37	1.24 (0.76-2.04)	0.39
CTLA-4 *(CTLA4*)	AA	Reference		Reference		reference	reference	reference	reference
rs4553808	AG	0.74 (0.45-1.22)	0.238	0.72 (0.43-1.20)	0.210	0.72 (0.42-1.22)	0.23	0.71 (0.42-1.21)	0.21
	GG	0.34 (0.15-0.74)	**0.007**	0.32 (0.15-0.72)	**0.006**	0.28 (0.11-0.68)	**0.01**	0.28 (0.11-0.67)	**0.01**
	AG + GG	0.64 (0.40-1.02)	0.062	0.62 (0.39-1.00)	0.051	0.61 (0.37-1.00)	0.05	0.61 (0.37-1.00)	0.05
CTLA-4 *(CTLA4*)	CC	Reference		Reference		reference	reference	reference	reference
rs5742909	CT	0.92 (0.48-1.73)	0.791	0.97 (0.51-1.84)	0.919	0.87 (0.45-1.68)	0.67	0.86 (0.44-1.68)	0.66
	TT	0.54 (0.12-2.43)	0.418	0.48 (0.10-2.24)	0.353	0.36 (0.64-2.00)	0.24	0.37 (0.07-1.05)	0.26
	CT+TT	0.867 (0.48-1.58)	0.640	0.90 (0.49-1.65)	0.736	0.80 (0.43-1.50)	0.49	0.80 (0.42-1.50)	0.48
CTLA-4 *(CTLA4*)	AA	Reference		Reference		reference	reference	reference	reference
rs231775	AG	1.25 (7.6-2.08)	0.328	1.27 (0.76-2.11)	0.368	1.21 (0.71-2.05)	0.49	1.22 (0.72-2.07)	0.47
	GG	0.94 (0.47-1.91)	0.869	0.97 (0.48-1.98)	0.933	0.74 (0.35-1.58)	0.44	0.76 (0.36-1.62)	0.48
	AG+GG	1.17 (0.73-1.86)	0.517	1.18 (0.74-1.89)	0.485	1.08 (0.66-1.76)	0.77	1.09 (0.67-1.77)	0.74

1 adj = adjustment for age CI = confidence interval; MM = malignant mesothelioma; OR = odds ratio; SNP = single nucleotide polymorphism

None of the investigated polymorphisms were associated with the risk of developing asbestosis (Supplementary Table 2).

We also evaluated the association between selected SNPs and risk of developing pleural plaques in comparison to control subjects exposed to asbestos but did not develop any asbestos-related disease (Table 3). Carriers of two polymorphic *CTLA4* rs4553808 G alleles had decreased risk of developing pleural plaques (OR = 0.28, 95% CI = 0.11-0.68, P = 0.01), the association was statistically significant also after age adjustment (OR = 0.28, 95% CI = 0.11-0.67, P = 0.01). The heterozygotes for this SNP were also less likely to develop pleural plaques (OR = 0.61, 95% CI = 0.37-1.00, P = 0.05), the results were borderline significant after age adjustment (OR = 0.61, 95% CI = 0.37-1.00, P = 0.05). No other SNP was significantly associated with susceptibility to pleural plaques.

We evaluated the association between selected SNPs and risk of developing MM in comparison to control group. Homozygotes for polymorphic *CTLA4* rs4553808 allele had decreased risk of developing MM (OR = 0.38, 95% CI = 0.16-0.93, P = 0.03), however, association was no longer statistically significant after adjustment for age (P_adj_ = 0.06). Moreover, in the dominant model, carriers of at least one polymorphic allele had a decreased risk for developing MM only after adjustment for age (OR = 0.56, 95% CI = 0.32-0.98, P = 0.04). No other SNP was significantly associated with susceptibility to MM (Table 4).

**TABLE 4.** Comparison of genotype frequencies distributions in patients with MM versus controls and versus patients with pleural plaques

Protein (Gene)	Genotype	MM versus controls	P	OR (95% CI)_adj_	P_adj_	MM versus pleural plaques	P	OR (95% CI)_α_adj	P_adj_
		OR (95% CI)				OR (95% CI)			
PD-1 (*PDCD1*)	CC	reference	reference	reference	reference	reference	reference	reference	reference
rs2227982	CT+TT	0.65 (0.2-2.113)	0.47	0.98 (0.26-3.7)	0.98	0.92 (0.56-1.27)	0.60	0.88 (0.36-2.16)	0.78
PD-1 (*PDCD1*)	CC	reference	reference	reference	reference	reference	reference	reference	reference
rs2227981	CT	1.30 (0.74-2.28)	0.36	1.33 (0.71-2.50)	0.38	0.93 (0.65-1.3)	0.67	0.93 (0.63-1.38)	0.72
	TT	1.02 (5.0-2.0)	0.95	0.92 (0.41-2.05)	0.84	0.89 (0.57-1.40)	0.61	0.81 (0.49-1.36)	0.43
	CT+TT	1.22 (0.72-2.05)	0.467	1.20 (0.67-2.17)	0.54	0.92 (0.66-1.27)	0.60	0.9 (0.62-1.30)	0.58
PD-1 (*PDCD1*)	GG	reference	reference	reference	reference	reference	reference	reference	reference
rs10204525	GA	1.09 (0.56-2.11)	0.80	0.80 (0.39-1.64)	0.55	0.76 (0.51-1.14)	0.18	0.81 (0.52-1.26)	0.35
	AA	1.02 (0.48-2.19)	0.94	0.35 (0.03-4.30)	0.41	2.0 (0.02-1.66)	0.14	0.32 (0.03-2.92)	0.31
	GA+AA	0.69 (0.37-1.28)	0.23	0.76 (0.38-1.52)	0.44	0.72 (0.49-1.07)	0.10	0.78 (0.40-1.20)	0.26
PD-L1 (*CD274*)	GG	reference	reference	reference	reference	reference	reference	reference	reference
rs2297136	GA	1.09 (0.56-2.12)	0.80	1.16 (0.55-2.41)	0.70	1.34 (0.89-2.01)	0.16	1.31 (0.83-2.08)	0.25
	AA	1.03 (0.48-2.19)	0.94	1.00 (0.43-2.34)	1.0	0.92 (0.59-1.46)	0.74	0.78 (0.47-1.32)	0.36
	GA+AA	1.07 (0.57-2.02)	0.83	1.11 (0.55-2.25)	0.78	1.18 (0.80-1.74)	0.40	1.10 (0.711-1.71)	0.66
PD-L1 (*CD274*)	GG	reference	reference	reference	reference	reference	reference	reference	reference
rs4143815	GC	1.23 (0.73-2.08)	0.44	1.27 (0.71-2.28)	0.42	0.99 (0.72-1.37)	0.95	0.96 (0.67-1.38)	0.83
	CC	1.16 (0.47-2.87)	0.74	0.93 (0.35-2.48)	0.90	0.60 (0.36-1.01)	0.05	0.6 (0.34-1.06)	0.08
	GC+CC	1.22 (0.74-2.01)	0.44	1.20 (0.69-2.10)	0.51	0.89 (0.66-1.21)	0.46	0.87 (0.62-1.23)	0.43
PD-L1 (*CD274*)	AA	reference	reference	reference	reference	reference	reference	reference	reference
rs4742098	AG	1.17 (0.68-2.00)	0.58	1.14 (0.63-2.06)	0.67	0.94 (0.68-1.30)	0.71	0.92 (0.64-1.32)	0.65
	GG	0.77 (0.31-1.94)	0.58	0.58 (0.21-1.60)	0.29	0.59 (0.33-1.05)	0.07	0.53 (0.28-1.01)	0.56
	AG + GG	1.08 (0.65-1.79)	0.76	1.01 (0.58-1.77)	0.97	0.87 (0.64-1.17)	0.35	0.83 (0.59-1.13)	0.30
CTLA-4 *(CTLA4*)	AA	reference	reference	reference	reference	reference	reference	reference	reference
rs4553808	AG	0.72 (0.42-1.24)	0.24	0.61 (0.33-1.13)	0.11	1.00 (0.72-1.40)	0.99	0.88 (0.60-1.29)	0.50
	GG	**0.38 (0.16-0.93)**	**0.03**	0.40 (0.15-1.06)	0.06	1.38 (0.66-2.86)	0.39	1.24 (0.56-2.73)	0.60
	AG+GG	0.64 (0.39-1.06)	0.08	**0.56 (0.32-0.98)**	**0.04**	1.04 (0.76-1.43)	0.79	0.92 (0.65-1.32)	0.66
CTLA-4 *(CTLA4*)	CC	reference	reference	reference	reference	reference	reference	reference	reference
rs5742909	CT	0.95 (0.48-1.86)	0.87	1.03 (0.48-2.22)	0.94	1.01 (0.73.-1.63)	0.67	1.11 (0.71-1.75)	0.64
	TT	0.57 (0.11-3.02)	0.51	0.31 (0.05-1.86)	0.20	1.59 (0.42-6.00)	0.49	1.42 (0.32-6.27)	0.65
	CT+TT	0.90 (0.47-1.70)	0.74	0.90 (0.44-1.86)	0.78	1.12 (0.76-1.66)	0.56	1.13 (0.73-1.76)	0.58
CTLA-4 *(CTLA4*)	AA	reference	reference	reference	reference	reference	reference	reference	reference
rs231775	AG	1.31 (0.757-2.25)	0.34	1.36 (0.74-2.50)	0.32	1.08 (0.78-1.50)	0.64	1.11 (0.77-1.61)	0.58
	GG	1.33 (0.63-2.81)	0.46	1.76 (0.76-4.08)	0.19	**1.79 (1.10-2.89)**	**0.02**	**2.1 (1.19-3.56)**	**0.01**
	AG + GG	1.31 (0.80-2.16)	0.29	1.47 (0.84-2.57)	0.18	1.22 (0.90-1.66)	0.21	1.29 (0.91-1.81)	0.15

1 adj = adjustment for age; CI = confidence interval; MM = malignant mesothelioma; OR = odds ratio; SNP = single nucleotide polymorphism

A comparison of patients with MM and pleural plaques showed that homozygotes for polymorphic *CTLA4* rs231775GG genotype were more susceptible to developing MM compared to subjects with pleural plaques (OR = 1.79, 95% CI = 1.10-2.89, P = 0.02). The association was still significant after adjustment for age (OR = 2.1, 95% CI = 1.19-3.56, P = 0.01). None of the other investigated polymorphisms showed any statistically significant associations (Table 3 and Table 4).

### *CTLA4* haplotypes distribution

Analysis of *CTLA4* haplotypes identified six SNP combinations. The most common haplotype was ACA (0.452), followed by ACG (0.327), GCA (0.113), GTA (0.103), ATA (0.003), and GTG (0.002). A comparison of predicted haplotype frequencies between subjects with any asbestos-related diseases and control group shows that subjects with haplotype GCA were less susceptible to any asbestos-related disease (OR = 0.49, 95% CI = 0.31-0.78, P = 0.003). The association remained statistically significant after adjustment for age (OR_adj_ = 0.46, 95% CI_adj_= 0.28-0.75, P_adj_ = 0.002). Other haplotypes did not show any significant associations (Table 5).

**TABLE 5.** Association of *CTLA4* haplotypes with susceptibility for asbestos diseases

Haplotype	Predicted frequency in asbestos diseases	Predicted frequency in controls	OR (95% CI)	P	OR (95% CI)_adj_	P_adj_
ACA	0.460	0.369	Reference			
ACG	0.328	0.320	0.84 (0.58-1.22)	0.359	0.84 (0.58-1.23)	0.379
GCA	0.106	0.183	0.49 (0.31-0.78)	**0.003**	0.46 (0.28-0.75)	**0.002**
GTA	0.101	0.120	0.68 (0.39-1.19)	0.181	0.71 (0.40-1.24)	0.222

1 adj = adjustment for age; CI = confidence interval; MM = malignant mesothelioma; OR = odds ratio; SNP = single nucleotide polymorphism

1 SNPs are listed from 5’ to 3’: rs4553808, rs5742909, rs231775

## Discussion

Our study assessed the association between genetic variability in three immune checkpoints: PD-1, PD-L1 and CTLA-4 and the risk of developing asbestos related diseases (pleural plaques, asbestosis and MM). The key finding in our study was the association of polymorphic *CTLA4* rs4553808 G allele with decreased risk of developing asbestos-related diseases of pleura, in particular MM and pleural plaques. However, carriers of polymorphic *CTLA4* rs231775 GG genotype had a higher risk of developing MM than the group of subjects with pleural plaques. None of investigated polymorphisms were associated with the risk of asbestosis.

Our study showed that the *CTLA4* rs4553808 G polymorphism had a protective role in development of pleural plaques and MM. This could be perhaps attributed to the fact that rs4553808 is located in the upstream regulatory region of *CTLA4* and may influence the binding site for TFs, but the consequences of such change in the binding side are still controversial. *In silico*, it was shown that change of A to G in position-1665 could create a new binding site for C/EBPβ TF.^25^ However, another study reported that the A to G substitution could lead to a loss of binding site for C/EBPβ TF.^26,27^ Therefore, the role of this SNP is still inconclusive and must be experimentally validated.

To the best of our knowledge there is no study that has evaluated associations between *CTLA4* rs4553808 and MM. However, it was widely studied in other types of cancer. Nevertheless, the results differ among studies. In a meta-analysis from 2020, it was shown that polymorphic *CTLA4* rs4553808 G allele significantly increased the risk of cancer in Asian population, but there were no significant associations observed in Caucasians.^28^ Therefore, the results of studies on Asian population are in agreement with hypothesis that A to G substitution leads to higher expression of *CTLA4*. However, another study reported that carriers of at least one A allele have a higher risk for development of gastric cancer.^29^ Consequently, that would be in agreement with hypothesis that rs4553808 downregulates *CTLA4* expression. Therefore, further studies are needed to evaluate the influence of rs4553808 on *CTLA4* expression.

Our study also showed the association of *CTLA4* rs231775 GA and GG genotype with increased risk of MM development when compared to pleural plaques. *CTLA4* rs231775 is a non-synonymous polymorphism which causes amino acid change from threonine (Thr) to alanine (Ala) in position 17 (p.Thr17Ala). It was reported that p.17Thr was associated with lower levels of T cell activation and proliferation.^28^ Moreover, lower levels of expression of *CTLA4* with p.17Ala were observed on the surface of T-cells. Therefore, *CTLA4* rs231775 AA genotype may be considered as a risk factor for cancer development.^28,30^ Indeed, *CTLA4* rs231775 was widely studied in different types of cancers, however, its influence on cancer risk is still controversial. It was shown that *CTLA4* rs231775 A allele increased the overall risk of cancer in Asian but not in Caucasian population.^30,31^ Two meta-analyses reported an increased risk of colorectal cancer in carriers of *CTLA4* rs231775 G allele.^32,33^ However, another meta-analysis showed that allele G plays a protective role in cancer development.^29^ A metaanalysis of several cancer types showed no evidence that *CTLA4* rs231775 would have an impact on lung cancer, and MM was not studied.^34^ Our results therefor warrant further research of the role of *CTLA4* rs231775 in the development of asbestos related diseases.

Our study observed no significant association between *CTLA4* rs5742909 and the risk of developing any asbestos disease. As this polymorphism is located in the promotor region, the T allele could have an impact on transcription factor LEF1 binding. As higher *CTLA4* mRNA expression levels were observed in T allele carriers, some studies concluded that this allele could be a risk factor for cancer.^28,35^ A meta-analysis showed association of rs5742909 T allele with increased risk of overall cancer susceptibility in both Asian and European population.^19^ However, *CTLA4* rs5742909 was not associated with risk for lung cancer.^36,37^

Our study did not show any statistically significant associations between *PDCD1* SNPs and susceptibility for developing any of investigated asbestos related diseases. *PDCD1* rs2227982 (c.644C>T, p.Ala215Val) is located in exon 5 and as it causes a nonsynonymous amino acid substitution in the extracellular domain of the PD-1, it could lead to a change in its structure and function.^38,39^ A meta-analysis performed in 2019 did not show a significant association between *PDCD1* rs2227982 and overall risk of cancer, however MM was not investigated.^18^ There was also no significant association between *PDCD1* rs2227982 and non-small cell lung cancer.^40^ Another investigated *PDCD1* polymorphism, rs2227981 (c.804T>C. p.Ala268=) in exon 5 leads to a synonymous amino acid substitution in PD-1. However, it was shown that PD-1 expression was lower in individuals with a more common rs2227981 genotype. Therefore, it was speculated that rs2227981 may be in linkage disequilibrium with another polymorphism influencing expression levels.^28,41^ Previous studies have reported that *PDCD1* rs2227981 TT genotype decreases the overall risk of cancer.^18,28^ However, no significant association were observed between *PDCD1* rs2227981 and many different types of cancer such as non-small cell lung cancer (NSCLC), colorectal cancer and breast cancer.^39^ The third investigated *PDCD1* polymorphism, rs10204525 (c.*889G>A) is located in a miRNA binding site in the 3’ untranslated region (3’UTR). According to one study, miRNA-4717 significantly decreased expression of the PD-1 in subjects with GG genotype, therefore it could have a protective role in cancer development.^28,42^ However, our results are in concordance with a meta-analysis did not find a significant association between *PDCD1* rs10204525 and the overall risk of cancer.^18^

Our study also did not show any significant association between any of the three investigated *CD274* SNPs: rs2297136, rs4143815, and rs4742098 and asbestos-related diseases. All of them are in 3’UTR region that it is crucial for post-translational modification.^28^ *In silico* prediction has shown that *CD274* rs2297136 (c.*93G>A) could affect the binding side for miRNA-296-5p and miRNA-324-5p.^43^ It was shown that expression of PD-L1 was inhibited in carriers of G allele. Therefore, G allele could play a protective role in cancer development.^28,43^ There have been many studies that investigate SNP *CD274* rs2297136 as risk factor for developing cancer. A previous study has shown that carriers of SNP *CD274* rs2297136 A allele had increased risk for developing NSCLC.^43^ Polymorphism *CD274* rs4143815 (c.*395G>C) is located in the binding site for miR-7-1*, miR-495, miR-298 and miR-570. As allele G changes binding site for miRNA-570, it could be cancer risk factor.^28,43^ According to the meta-analysis, *CD274* rs4143815 was associated with increased overall risk of cancer.^44^ There was no association between *CD274* rs4143815 and risk of developing NSCLC.^44^ Polymorphism *CD274* rs4742098 (c.*2635A>G) is located in the binding site for miRNA-138. Allele A increases the capability of binding miRNA-138 and could therefore be protective for cancer development.^28,43^ Carriers of *CD274* rs4742098 G allele had significantly higher risk of developing NSCLC, according to one study.^43^

The limitation of our study is a small control group. However, the strength of our study is its relatively large and well-defined sample. All subjects were from genetically homogenous Central Europe Caucasian populations, therefore there are not a lot of other variables that could influence our analysis. Another limitation of our study was that the group of MM patients was significantly older than other groups. This finding is in agreement with some other studies, probably due to the long latency period between asbestos-exposure and MM development.^2,45^ Therefore, we performed multivariable analysis with adjustment for age.

In conclusion, our data indicate that *CTLA4* polymorphisms may have an impact on the risk of asbestos-diseases of the pleura such as pleural plaques and MM. Therefore, future studies should investigate if these polymorphisms may also play a role in the outcome of MM treatment with immunotherapy.

## Supplementary Material

Supplementary Material Details
