# Supplementary material for: Genetic variability of immune checkpoints in asbestos-related diseases
Source: Radiol Oncol. 2026 Jun 26;60(2):271–81. doi: 10.2478/raon-2026-0035 (PMC13307012; doi:10.2478/raon-2026-0035)
Supplement: Supplementary file 1 — Supplementary Material Details [file raon-2026-0035_sm.pdf]

Genetic variability of immune checkpoints in asbestos-related diseases

Irma Zeljkovic, Katja Goricar, Tanja Blagus, Alenka Franko, Viljem Kovac, Vita Dolzan

Radiol Oncol 2026; 60(2): 271-281.

doi: 10.2478/raon-2026-0035

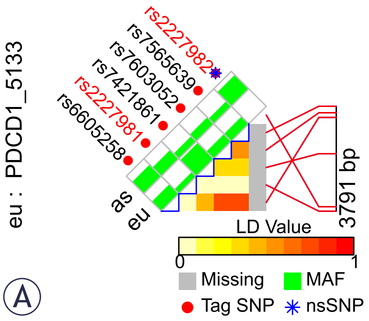

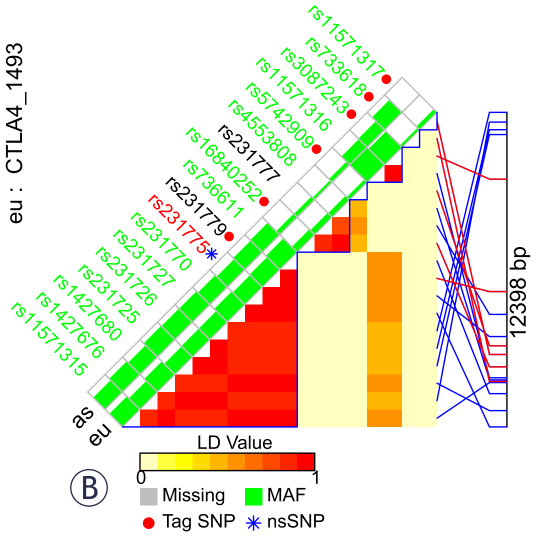

SUPPLEMENTARY FIGURE 1. Tag single nucleotide polymorphism (tagSNP) analysis of the immune checkpoint genes.

(A) ***PDCD1*.** 6 SNPs were identified within the gene region: 2 were located in the coding region (rs numbers in red), while 4 were in the intron region (rs numbers in black). Based on the rare allele frequencies reported in the data base SNP (dbSNP) and the function prediction rs2227982, rs2227981 and rs10204525 were included in the study.

(B) ***CTLA4*.** 18 SNPs were identified within the gene region: 1 was in the coding region (rs numbers in red), while the others were located near the gene (rs numbers in green). Based on the rare allele frequencies reported in the dbSNP database and the function prediction 5 SNPs were selected. Using the LDlink tool we established that rs231775 and rs231779 are in linkage disequilibrium (LD); therefore, only rs231775 was included in our study. Furthermore, rs16840252 and rs4553808 were in LD; therefore, we included only rs4553808 as it had been more frequently studied. We also included rs5742909 SNP as it was in the coding region.

(C) ***CD274*.** 43 SNPs were identified within the gene region: 2 in the 3'-UTR (rs numbers in blue), 6 in the near-gene region (rs numbers in green), and the rest in the introns (rs numbers in black). Based on the rare allele frequencies reported in the dbSNP database and the function prediction rs4143815, rs4742098, and rs2297136 were included in the study.

MAF = minor allele frequency;
nsSNP = nonsynonymous single nucleotide polymorphism

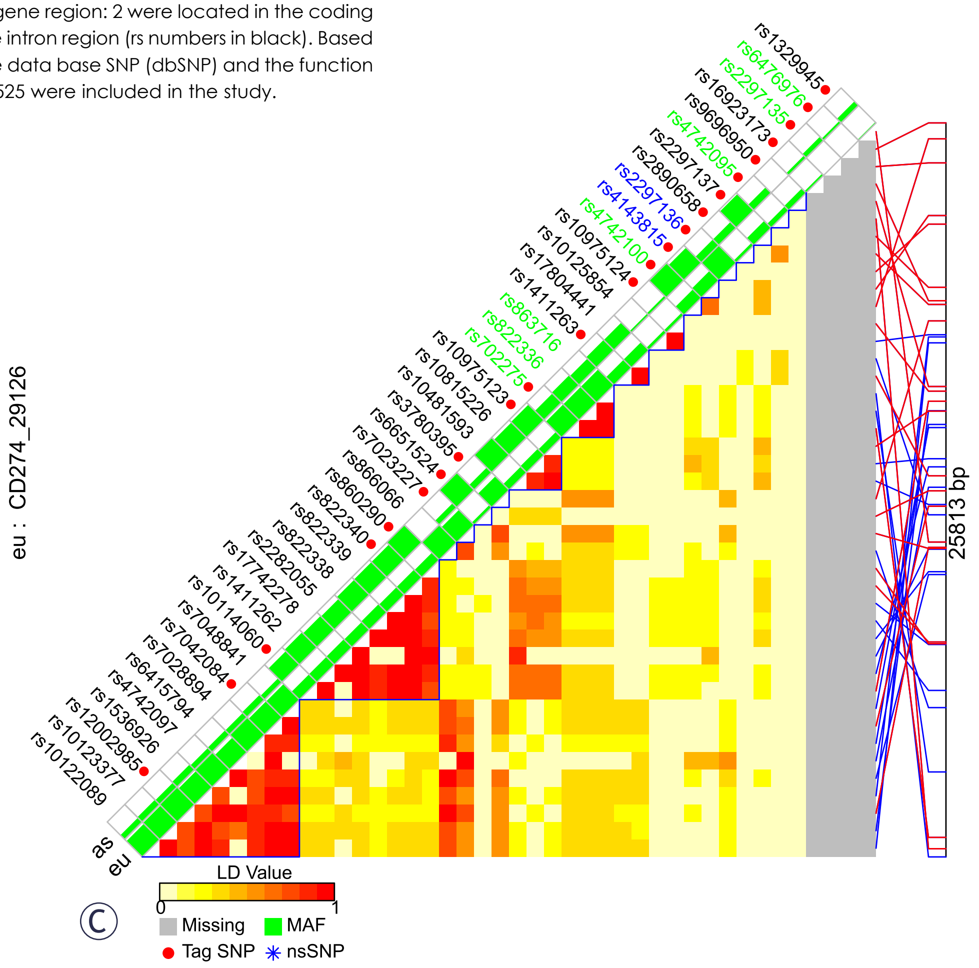

SUPPLEMENTARY TABLE 1. Selected *PDCD1, CD274* and *CTLA4* polymorphisms and their predicted role

Gene	Polymorphism	Nucleotide change	MAF (European)	Predicted role
PDCD1	**rs2227982**	**C/T**	**A = 0.011**	**Missense mutation**
PDCD1	**rs2227981**	**C/T**	**A = 0.433**	**Silent mutation**
PDCD1	rs11568821	C/T	T = 0.087	Mutation in an intronic region
PDCD1	**rs10204525**	**G/A**	**T = 0.100**	**In the 3'-UTR; may affect miRNA binding**
CD274	**rs4143815**	**G/C**	**C = 0.289**	**In the 3'-UTR; may affect miRNA binding**
CD274	**rs4742098**	**A/G**	**G = 0.238**	**In the 3'-UTR; may affect miRNA binding**
CD274	**rs2297136**	**G/A**	**G = 0.474**	**In the 3'-UTR; may affect miRNA binding**
CTLA4	rs733618	T/C	C = 0.077	Affects TF binding
CTLA4	rs11571317	C/T	T = 0.085	Affects TF binding
CTLA4	**rs231775**	**A/G**	**G = 0.347**	**Missense mutation; affects splicing**
CTLA4	**rs5742909**	**C/T**	**T = 0.093**	**Affects TF binding**
CTLA4	rs16840252	C/T	T = 0.175	Affects TF binding
CTLA4	rs3087243	A/G	A = 0.454	/
CTLA4	**rs4553808**	**A/G**	**G = 0.121**	**Affects TF binding**
CTLA4	rs231779	C/T	T = 0.372	/

MAF = minor allele frequency; TF = transcription factor

Selected polymorphisms are bolded.

SUPPLEMENTARY TABLE 2. Association of selected single nucleotide polymorphisms (SNPs) with susceptibility to asbestosis compared to controls

Protein (Gene)	SNP	Genotyp	OR (95% CI)	P	OR (95% CI)$_{adj}$	P$_{adj}$
PD-1 (*PDCD1*)	rs2227982	CC	reference	reference	reference	reference
		CT+TT	0.90 (0.26–3.17)	0.87	1.02 (0.28–3.70)	0.98
PD-1 (*PDCD1*)	rs2227981	CC	reference	reference	reference	reference
		CT	1.10 (0.56–2.03)	0.77	1.04 (0.56–1.95)	0.90
		TT	0.86 (0.39–1.90)	0.72	0.82 (0.37–1.84)	0.64
		CT+TT	1.03 (0.58–1.82)	0.93	0.97 (0.54–1.75)	0.93
PD-1 (*PDCD1*)	rs10204525	GG	reference	reference	reference	reference
		GA	0.98 (0.49–1.97)	0.96	0.82 (0.4–1.70)	0.60
		AA	0.25 (0.02–2.77)	0.26	3.00 (0.03–3.46)	0.33
		GA+AA	0.90 (0.46–1.75)	0.75	0.77 (0.38–1.53)	0.45
PD-L1 (*CD274*)	rs2297136	GG	reference	reference	reference	reference
		GA	0.61 (0.30–1.24)	0.17	0.52 (0.25–1.09)	0.08
		AA	0.70 (0.31–1.55)	0.37	0.61 (0.27–1.39)	0.24
		GA+AA	0.90 (0.46–1.75)	0.19	0.55 (0.28–1.10)	0.09
PD-L1 (*CD274*)	rs4143815	GG	reference	reference	reference	reference
		GC	1.24 (0.69–2.21)	0.47	1.20 (0.66–2.18)	0.54
		CC	1.71 (0.66–4.42)	0.27	1.92 (0.73–5.07)	0.19
		GC+CC	1.32 (0.77–2.29)	0.32	1.33 (0.76–2.33)	0.32
PD-L1 (*CD274*)	rs4742098	AA	reference	reference	reference	reference
		AG	1.34 (0.75–2.41)	0.33	1.35 (0.74–2.45)	0.33
		GG	0.81 (0.29–2.28)	0.69	0.87 (0.30–2.51)	0.80
		AG+GG	1.23 (0.7–2.14)	0.46	1.25 (0.71–2.20)	0.43
CTLA4	rs4553808	AA	reference	reference	reference	reference
		AG	0.82 (0.45–1.47)	0.50	0.83 (0.45–1.51)	0.54
		GG	0.39 (0.14–1.09)	0.07	0.44 (0.16–1.25)	0.13
		AG+GG	0.71 (0.41–1.24)	0.23	0.74 (0.42–1.29)	0.29
CTLA4	rs5742909	CC	reference	reference	reference	reference
		CT	0.99 (0.48–2.07)	0.99	1.17 (0.55–2.50)	0.69
		TT	0.92 (0.16–5.20)	0.93	0.87 (0.15–4.93)	0.88
		CT+TT	0.98 (0.49–1.97)	0.97	1.12 (0.55–2.30)	0.75
CTLA4	rs231775	AA	reference	reference	reference	reference
		AG	1.28 (0.71–2.30)	0.42	1.28 (0.70–2.35)	0.42
		GG	0.75 (0.31–1.79)	0.52	0.83 (0.34–2.03)	0.68
		AG+GG	1.13 (0.65–1.95)	0.67	1.16 (0.66–2.03)	0.61

adj = adjustment for age; CI = confidence interval; OR = odds ratio; PD-1 = programmed cell death receptor 1; PD-L1 = programmed cell death ligand-1
